# Synthesis and Characterization of ZnO-TiO_2_/Carbon Fiber Composite with Enhanced Photocatalytic Properties

**DOI:** 10.3390/nano10101960

**Published:** 2020-10-01

**Authors:** Bishweshwar Pant, Gunendra Prasad Ojha, Yun-Su Kuk, Oh Hoon Kwon, Yong Wan Park, Mira Park

**Affiliations:** 1 Carbon Composite Energy Nanomaterials Research Center, Woosuk University, Wanju-Gun, Jeollabuk-do 55338, Korea; bisup@jbnu.ac.kr (B.P.); gpojha10@gmail.com (G.P.O.); 2 Korea Institute of Carbon Convergence Technology (KCTECH), Jeonju 54853, Korea; yunsu@kctech.re.kr; 3 Research and Development Division, Korea Institute of Convergence Textile, Iksan 54588, Korea; simulation@kictex.re.kr (O.H.K.); pywspirit@kictex.re.kr (Y.W.P.)

**Keywords:** electrospinning, hydrothermal, ZnO-TiO_2_-carbon, photocatalyst

## Abstract

Herein, we prepared a novel photocatalytic ZnO-TiO_2_ loaded carbon nanofibers composites (ZnO-TiO_2_-CNFs) via electrospinning technique followed by a hydrothermal process. At first, the electrospun TiO_2_ NP-embedded carbon nanofibers (TiO_2_-CNFs) were achieved using electrospinning and a carbonization process. Next, the ZnO particles were grown into the TiO_2_-CNFs via hydrothermal treatment. The morphology, structure, and chemical compositions were studied using state-of-the-art techniques. The photocatalytic performance of the ZnO-TiO_2_-CNFs composite was studied using degrading methylene blue (MB) under UV-light irradiation for three successive cycles. It was noticed that the ZnO-TiO_2_-CNFs nanocomposite showed better MB removal properties than that of other formulations, which might be due to the synergistic effects of carbon nanofibers and utilized metal oxides (ZnO and TiO_2_). The adsorption characteristic of carbon fibers and matched band potentials of ZnO and TiO_2_ combinedly help to boost the overall photocatalytic performance of the ZnO-TiO_2_-CNFs composite. The obtained results from this study indicated that it can be an economical and environmentally friendly photocatalyst.

## 1. Introduction

Water pollution caused by the presence of organic pollutants has detrimental effects on human beings and aquatic life. Photocatalytic degradation has been applied as the most promising method to solve the organic pollutant problem [1,2,3,4]. Therefore, the development of an effective material with simultaneous adsorption and photocatalytic dye degradation properties is of great importance in environmental protection [5,6]. Semiconductor photocatalysis has attracted intense attention as an effective technology in the field of pollution removal [7,8,9,10,11]. Among various semiconductor photocatalytic materials, titanium dioxide (TiO_2_) and zinc oxide (ZnO) have been widely studied due to their good photocatalytic activities, stability, low-cost, and nontoxicity [12,13,14,15]. However, the wide bandgap (TiO_2_: 3.2 eV and ZnO: 3.3 eV), high recombination of photogenerated charge carriers, and poor adsorption property are major drawbacks that limit the practical application of TiO_2_ and ZnO [16,17]. Due to their wide band gaps, they are active only in the UV-light region, which is less than 5% of the solar light spectrum. In addition, the high recombination of electron-hole pairs of ZnO and TiO_2_ leads to a low photon-to-electron conversion efficiency [18,19,20,21,22]. Therefore, improving photocatalytic activities via modification has become an important task.

Previous studies revealed that incorporating ZnO and TiO_2_ into a hybrid composite structure could improve their photocatalytic properties [12,23,24,25]. The coupling of TiO_2_ with ZnO suppresses the electron-hole recombination rate, and consequently, higher photocatalytic efficiency can be achieved [12,26]. However, preparing such a heterostructure material in nanoscale is still challenging because of the structural complexity and difficulties in controlling the crystal growth of the materials. In addition, despite increasing the surface area and enhancing the photocatalytic activity, the nanostructured photocatalysts may create a secondary pollution problem due to the difficulty in separation after use [19,27]. Moreover, the agglomeration of nano-sized particles is another issue in the widespread application of photocatalyst in wastewater treatment [28]. In order to solve the agglomeration and secondary pollution, recently our group has developed various photocatalytic particles immobilized into the carbon nanofibers using electrospinning and hydrothermal methods [13,19,29,30,31]. The carbon fiber not only provides support to deposit the photocatalytic materials on its surface but also improves the overall photocatalytic efficiency of the composite by enhancing the conductivity and adsorptivity [12,29,32,33].

In recent years, electrospinning technology has made a significant contribution in various applications, including photocatalysis [34,35,36,37,38]. Recently, our group prepared titania nanoparticles (TiO_2_ NPs) incorporated into carbon nanofibers (TiO_2_-CNFs) using an electrospinning process followed by suitable thermal treatment [19,29,39]. The assembly of ZnO into the TiO_2_-CNFs can further enhance the photocatalytic activity of the resulting composite; however, the deposition of ZnO particles into the carbon fibers using a single-step approach is challenging due to the instability of ZnO at a higher temperature [40,41]. On the basis of the above understandings, herein we have synthesized ZnO-TiO_2_ decorated carbon nanofiber composite using a two-step route for methylene blue (MB) degradation. In the first step, TiO_2_ NP-embedded carbon nanofibers (TiO_2_-CNFs) were prepared using electrospinning and calcination processes. Next, the TiO_2_-CNFs served as a substrate to grow ZnO particles, and ZnO-TiO_2_ decorated carbon fiber composite (ZnO-TiO_2_-CNFs) was obtained using the hydrothermal method. The ZnO-TiO_2_-CNFs composite showed better performance than that of the individual materials. The enhanced photocatalytic and antibacterial activities in the composite photocatalyst is due to the enhanced surface area and the combined effects of carbon nanofiber and the matched bandgap of ZnO-TiO_2_.

## 2. Materials and Methods

### 2.1. Materials

Polyvinylpyrrolidone (PVP, average molecular weight ~130000 via LS), titanium tetraisopropoxide (TTIP, 97 %), ethanol, acetic acid, zinc nitrate hexadydrate, bishexamethylene tetramine, and methylene blue trihydrate (MB) were purchased from Sigma-Aldrich, St. Louis, USA. All the chemicals were used as received.

### 2.2. Fabrication of TiO_2_-CNFs Composite

At the beginning, 1.5 g of TTIP and 3 g of acetic acid were stirred in a vial for 10 min. Then, 0.5 g of PVP and 4 g of ethanol were added to the TTIP/acetic acid mixture and vigorously stirred for 6 h. Next, the electrospinning was performed at 20 kV maintaining the tip of the capillary-to-collector distance at 15 cm. The collected nanofiber membrane was carbonized under an argon atmosphere at 800 °C for 3 h. During the carbonization process, the heating rate was maintained at 3 °C/min. For comparison, TiO_2_ nanofibers were also prepared. To prepare TiO_2_ nanofiber, the as-obtained nanofiber membrane from electrospinning was subjected to calcination in an air atmosphere at 600 °C for 2 h.

### 2.3. Fabrication of ZnO-TiO_2_-CNFs Composite

The TiO_2_-CNFs composite fiber obtained using electrospinning was used as a substrate to grow the ZnO NPs over its surface. Briefly, 10 mg of TiO_2_-CNFs composite was sonicated into 35 mL of double distilled water for 1 h. Next, 0.7 g of zinc nitrate hexahydrate and 0.5 g of bishexamethylene triamine were added to the mixture and stirred for 6 h. Then, the hydrothermal treatment was carried out at 150 °C for 1 h. Finally, the product was filtered and washed with water and ethanol several times and dried at 80 °C for 12 h. For comparison, pristine ZnO particles were also prepared using the hydrothermal method under the identical condition without using TiO_2_-CNFs.

### 2.4. Characterization

A wide-angle X-ray diffractometer (XRD, Rigaku Co., Tokyo, Japan) was used to analyze the crystallinity of the samples. Field emission scanning electron microscopy (FESEM, Hitachi, Japan) was used to investigate the morphology of the synthesized photocatalysts. The adsorption spectra were observed using a UV-visible spectrometer (Lambda 900, Perkin Elmer, USA). A PerkinElmer instrument (LS-55) was used for photoluminescence (PL) spectra.

### 2.5. Phtocatalytic Degradation of MB

The photocatalytic behavior of the pristine TiO_2_ NFs, TiO_2_-CNFs, and ZnO-TiO_2_-CNFs was tested by analyzing the degradation of MB aqueous solution under UV irradiation as explained in our previous experiment [12]. Briefly, 20 mg of catalyst was taken along with 25 mL of 10 ppm MB solution in a glass vial and kept in a dark condition for 30 min. An ultraviolet lamp (λ = 365 nm) was used as a light source. The distance from the lamp to the dye solution was 5 cm. The treated MB was collected at specific time intervals and centrifuged to separate the photocatalyst particles. The absorbance intensity of the supernatant was measured using a UV-vis spectrophotometer. For the cyclic reusability test, the experiment was carried out for three cycles in accordance with our previous experiments [12,19]. In reusability experiments, after each cycle, the catalyst was filtered and fresh MB solution with the same concentration was utilized. The experiment was carried out at identical conditions. The MB removal efficiency was calculated using the given equation:

(1) $$MB\;removal\;efficiency\left(\%\right)=\frac{\left(C-C_{o}\right)}{C}\times 100$$

where C and C_o_ are the initial and final concentration of MB.

## 3. Results and Discussion

**Figure 1:**
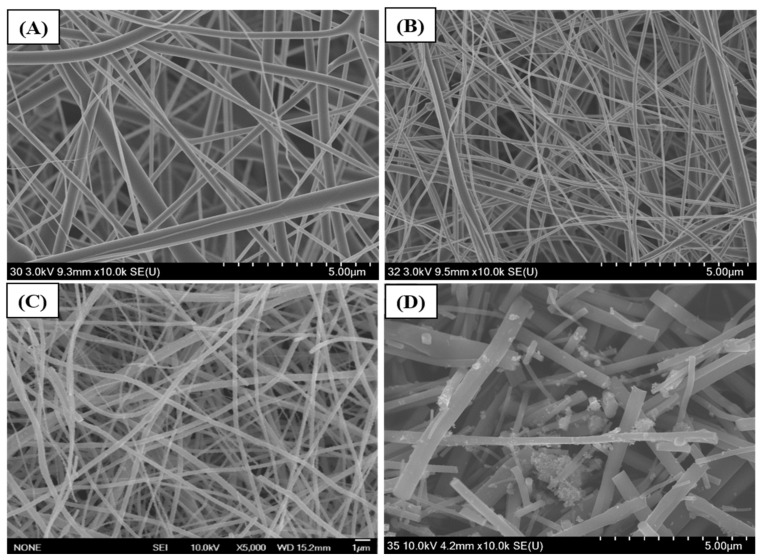
Field emission scanning electron microscopy (FESEM) images of titanium tetraisopropoxide/ polyvinylpyrrolidone (TTIP/PVP) nanofibers (**A**), titanium dioxide (TiO_2_) NFs (**B**), TiO_2_-carbon nanofibers (CNFs) (**C**), and zinc oxide (ZnO)-TiO_2_-CNFs (**D**).

**Figure 2:**
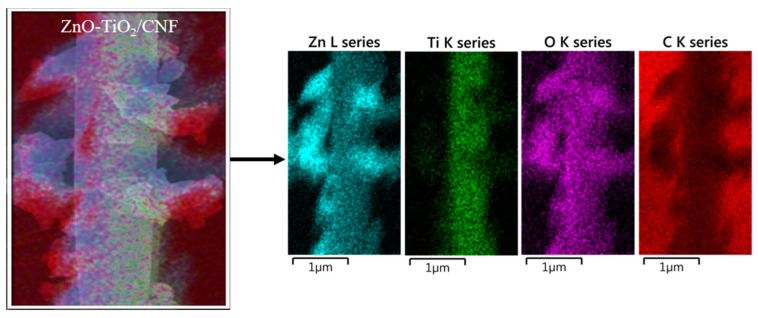
Elemental mapping of the as-prepared ZnO-TiO_2_-CNFs.

**Figure 3:**
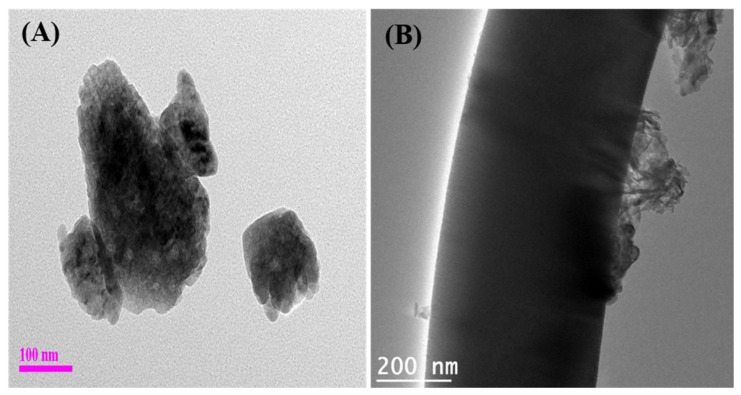
TEM image of ZnO particle (**A**) and ZnO-TiO_2_-CNFs composite (**B**).

FESEM was used to investigate the surface morphology of the TiO_2_, TiO_2_-CNFs, and ZnO-TiO_2_/CNFs composite fibers (Figure 1). The nanofibers obtained via the electrospinning of the TTIP/PVP solution showed a smooth and continuous fibrous morphology without beads (Figure 1A). When the TTIP/PVP nanofiber mat was calcined at 600 °C for 2 h in air atmosphere, the PVP was removed and TiO_2_ NFs were obtained (Figure 1B). On the other hand, when the TTIP/PVP nanofiber mat underwent a direct carbonization process at 800 °C for 3 h under an inert atmosphere of argon gas, the titanium tetraisopropoxide decomposed to TiO_2_ and the PVP was converted to carbon, thereby leading to the formation of TiO_2_ NP-embedded carbon nanofibers (Figure 1C). It is important to note that the fibrous morphology was preserved after the thermal treatment in both cases. Next, the hydrothermal method was employed to load ZnO NPs on the surface of the TiO_2_-CNFs composite. As in Figure 1D, the ZnO NPs are attached on the surface of TiO_2_-CNFs with good distribution. The existence of ZnO, TiO_2_, and carbon in the composite was confirmed by elemental mapping. As in Figure 2, the ZnO-TiO_2_-CNFs composite showed the presence of Zn, Ti, O, and C. To examine the assembly of ZnO particles in the TiO_2_-CNFs composite, TEM analysis was carried out. Figure 3A shows the TEM image of ZnO particles obtained using hydrothermal synthesis. The size of the ZnO particles was recorded as 100–400 nm. It should be noted that when the ZnO particles were loaded into the TiO_2_-CNFs composite, the size of the ZnO particles was reduced to 50–200 nm in the resulting composite (Figure 3B). We observed the same results in our previous publication also [12]. When the ZnO particles were loaded into the TiO_2_-CNFs, the presence of TiO_2_-CNFs could hinder the aggregation of crystallized ZnO particles during the hydrothermal treatment. Furthermore, the addition of TiO_2_-CNFs into the same volume of ZnO precursor suspension decreased the concentration of ZnO precursor per unit volume, and consequently, the size of the particles was reduced in the ZnO-TiO_2_-CNFs composite. Furthermore, the presence of TiO_2_ and ZnO along with the carbon fibers was also confirmed using TEM-EDX. A TEM-EDX in a randomly chosen line detected all utilized materials (Figure S1).

**Figure 4:**
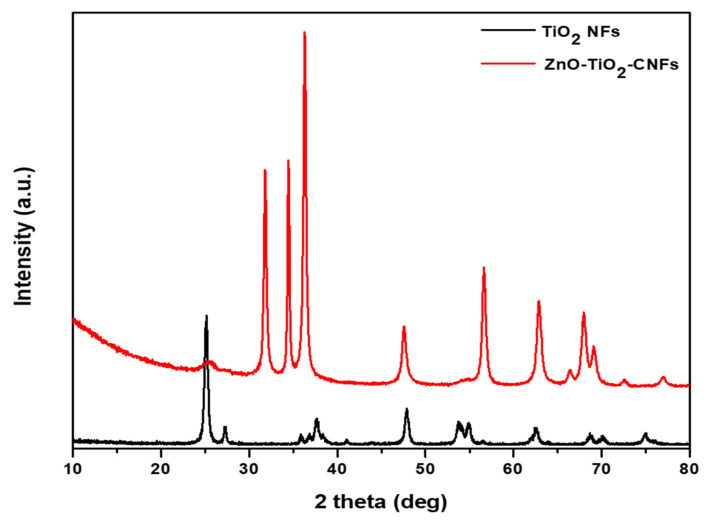
XRD spectra of TiO_2_ NFs and ZnO-TiO_2_-CNFs composite.

Figure 4 displays the XRD spectra of various samples. The XRD spectra of TiO_2_ NFs showed several peaks at 2θ values of 27.35°, 36.0°, 41.2°, 54.25°, 56.55°, 62.75°, 63.9°, and 68.95°, which correspond to (1 1 0), (1 0 1), (1 1 1), (2 1 1), (2 2 0), (0 0 2), (3 1 0), and (3 0 1) crystal plane of rutile, respectively [31,42]. Similarly, the peaks at 25°, 38°, and 47.89° matched with the crystal planes of (1 0 1), (0 0 4), and (2 0 0) anatase TiO_2_, respectively [31,43]. After loading ZnO NPs using the hydrothermal process, the resulting ZnO-TiO_2_-CNFs composite showed TiO_2_ and ZnO peaks. Additionally, a broad peak was observed at 2θ values of 25°, which is due to the carbon structure [13]. In our previous study, we found that the anatase phase was transformed to rutile at a calcination temperature above 800 °C [39]. Since the TiO_2_ NFs with both anatase and rutile phase show better photocatalytic properties than that of either phase alone [44], calcination was performed at 800 °C to maintain the phase crystallinity of the composite structure. The presence of ZnO in the ZnO-TiO_2_-CNFs composite was confirmed by several peaks at 31.8°, 34.5°, 36.4°, 47.6°, 56.6°, 62.8°, 66.3°, 68.1°, 69°, and 76.88°, which correspond to the crystal planes of (1 0 0), (0 0 2), (1 0 1), (1 0 2), (1 1 0), (1 0 3), (2 0 0), (1 1 2), (2 0 1), and (2 0 2) phases of ZnO [12,13,45]. The presence of TiO_2_, ZnO, and carbon peaks in the ZnO-TiO_2_-CNFs composite indicated that the ZnO NPs were successfully loaded into the TiO_2_-CNFs composite.

**Figure 5:**
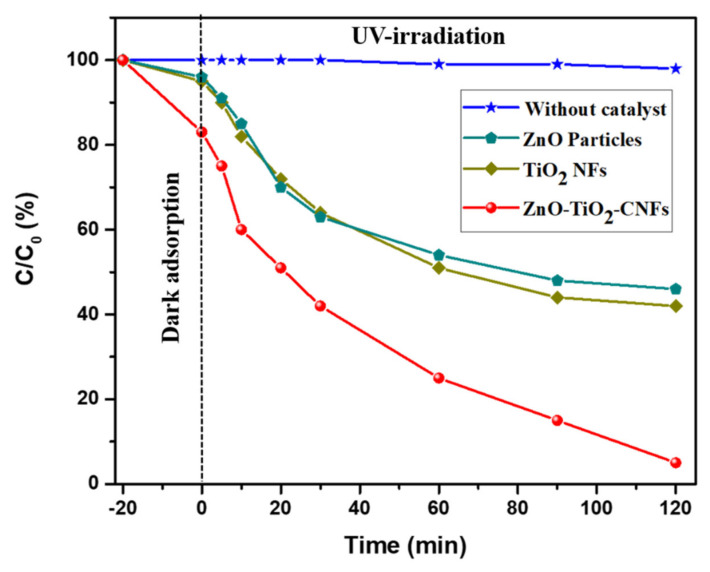
Photocatalytic methylene blue (MB) degradation using various photocatalysts under UV irradiation.

**Figure 6:**
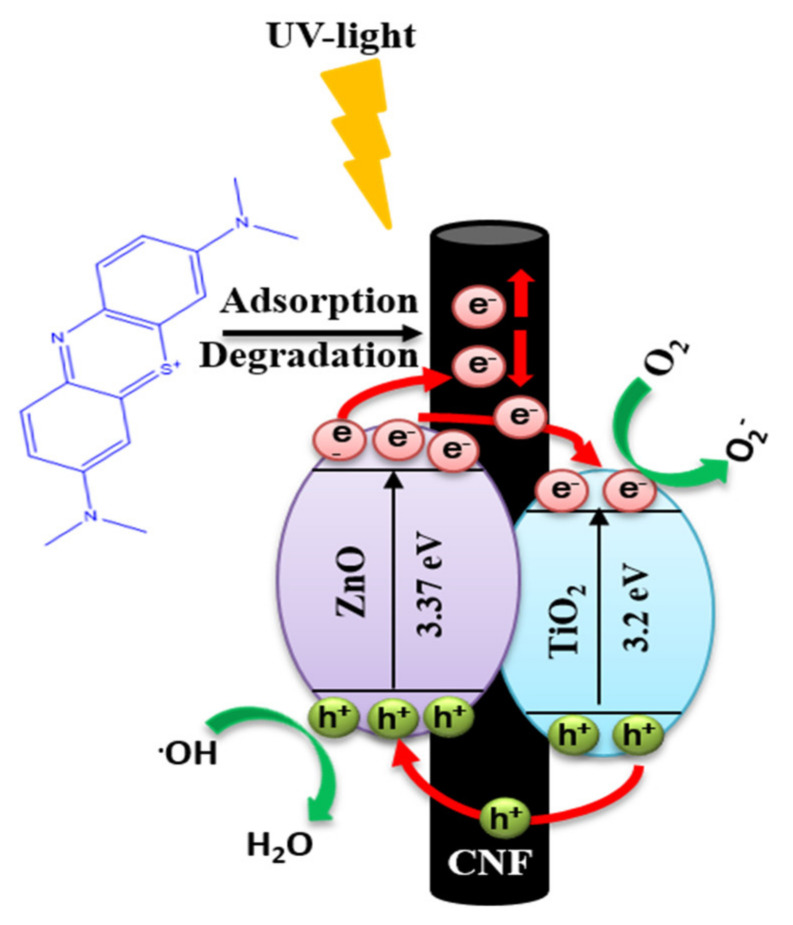
Proposed mechanism of photocatalysis by the as-prepared ZnO-TiO_2_-CNFs composite photocatalyst.

Figure 5 represents the photocatalytic degradation of MB aqueous solution by the various photocatalysts under UV irradiation. Since the MB may degrade due to the UV alone, a blank test was performed to monitor the self-degradation under UV irradiation. The blank test under UV light irradiation without using any photocatalysts showed about 2% degradation after 2 h. This means that the MB solution underwent negligible self-degradation using UV light during the photocatalytic test. The TiO_2_ NFs showed slightly better photocatalytic performance than that of the ZnO particles. It was observed that the MB removal was higher in the case of ZnO-TiO_2_-CNFs than that of the other formulations. The enhancement in the photocatalytic performance of the ZnO-TiO_2_-CNFs as compared to the TiO_2_ NFs indicated that the introduction of ZnO played an important role in the photocatalysis. A schematic diagram showing the proposed mechanism of photocatalysis is given in Figure 6. The known adsorption property of carbon fibers and the coupling effect of the TiO_2_ and ZnO are the two important factors for a pronounced higher photocatalytic property in ZnO-TiO_2_-CNFs composite photocatalyst [12,46]. In the ZnO-TiO_2_-CNFs composite photocatalytic system, the adsorption and degradation processes take place simultaneously. First, the MB molecules are adsorbed at the surface of the photocatalyst, and under the UV light irradiation, the photogenerated electrons are transferred from the conduction band of excited ZnO to the conduction band of TiO_2_. Moreover, the photogenerated holes enter the valence band of ZnO from the valence band of TiO_2_ [12,14]. In addition, the carbon fiber also possesses a conductive property. Such kinds of transfer of charge carriers helps in charge separation, thereby enhancing the overall photocatalytic performance [47]. The electrons react with the dissolved oxygen molecules to produce oxygen peroxide radicals (O_2_−) whereas the holes react with OH^−^ (derived from H_2_O) to produce hydroxyl radicals (OH). Both peroxide and hydroxide radicals degrade the MB dye molecules photocatalytically into CO_2_ and H_2_O [6].

The reusability of the prepared photocatalyst was examined via the cyclic experiments for three cycles (Figure S2). For this purpose, the used photocatalyst was separated and applied for photodegradation under similar conditions. It was noticed that the MB removal efficiency of the ZnO-TiO_2_-CNFs photocatalyst was slightly reduced in the successive cycles whereas the TiO_2_ NFs showed drastic loss in the photocatalytic activities in the successive cycles. The slight loss in the MB photocatalytic activity of the ZnO-TiO_2_-CNFs composite may be due to the loss in the adsorption property of the carbon [12].

**Figure 7:**
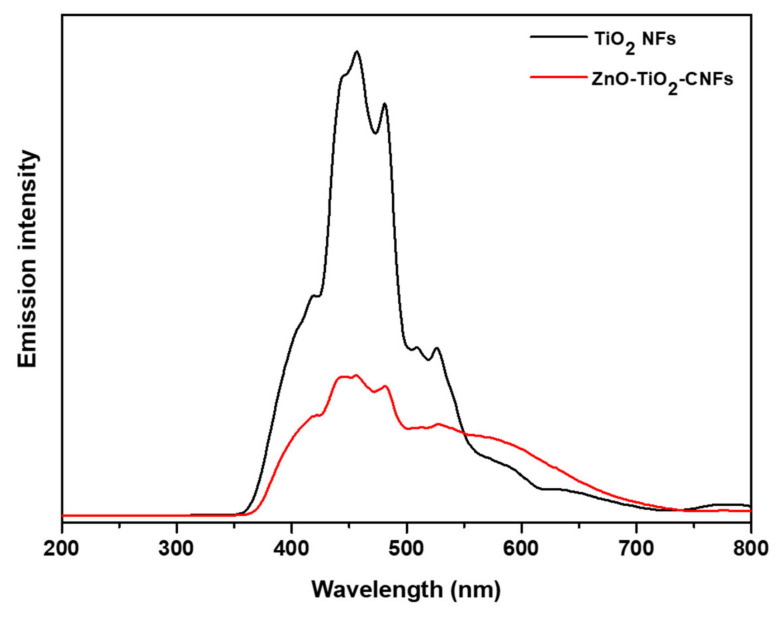
Photoluminescence (PL) spectra of ZnO-TiO_2_-CNFs composite as compared to the pristine TiO_2_ NFs.

In order to confirm that the loading of ZnO into the TiO_2_-CNFs composite results in the suppression of the rate of recombination of electron-hole (e–h), we studied the PL spectra of the photocatalysts (Figure 7). The samples were excited at the wavelength of 325 nm at room temperature. After the deposition of ZnO NPs via the hydrothermal process, the PL intensity decreased remarkably as compared to the pristine TiO_2_ NFs, which reveals the efficient charge transport at the interface between ZnO-TiO_2_ at the body of carbon fibers. The suppression in the PL intensity is the indication of the lower charge recombination rate and higher carrier transport efficiency [6]. Therefore, we obtained better performance using ZnO-TiO_2_-CNFs for MB removal than when using TiO_2_ NFs alone.

**Figure 8:**
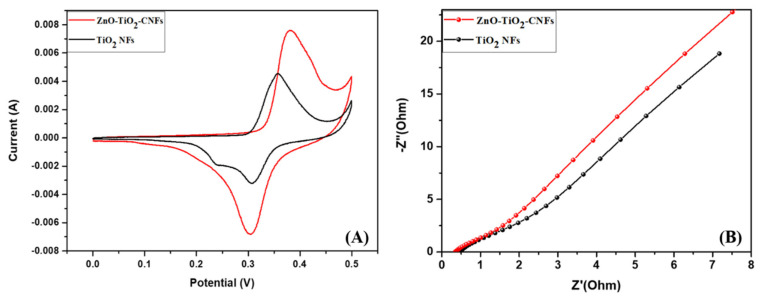
Cyclic voltammetry (CV) curves at the scan rate of 10 mV/s (**A**) and EIS spectra (**B**) of ZnO-TiO_2_-CNFs composite as compared to TiO_2_ NFs.

We studied the electrochemical performance of the as-prepared photocatalysts using cyclic voltammetry (CV) on a Versastat3 using a three-electrode system in a 2 M KOH solution. A slurry was prepared by mixing photocatalyst (80%), carbon black (10%), and polyvinyl fluoride (10%) in N-methyl-2-pyrrolidone (NMP) and then loaded into a nickel foam. The active material-loaded nickel foam was considered as a working electrode whereas the pt wire and Ag/AgCl were used as the counter and the reference electrode, respectively. The CV was recorded within the potential range of 0 to 0.5 V. Figure 8A gives the CV results of the pristine TiO_2_ NFs and ZnO-TiO_2_-CNFs composite. As in the figure, the higher catalytic activity of the ZnO-TiO_2_-CNFs composite is verified by the higher oxidation and reduction peaks and higher area under the CV curves as compared to that of the TiO_2_ NFs [6]. The slight difference in the oxidation-reduction peaks of the ZnO-TiO_2_-CNFs composite fibers compared to that of the TiO_2_ NFs is due to the structural difference. The pristine TiO_2_ fiber is composed of both rutile and anatase phase, whereas in ZnO-TiO_2_-CNFs the rutile phase is dominant. In addition, both ZnO and TiO_2_ in the composite contributed to the pseudocapacitive behavior [6]. The presence of carbon in the composite also influences the electrochemical properties [39]. The charge transfer resistance was studied by electrochemical impedance spectroscopy (EIS) (Figure 8B). The ZnO-TiO_2_-CNFs revealed the smallest arc radius at the high frequency region as compared to the TiO_2_ NFs. Since the arc is related to the charge transfer resistance, it can be concluded that the ZnO-TiO_2_-CNFs have the lower charge transfer resistance or higher conductivity as compared to the pristine TiO_2_ NFs [6]. The finding from CV, EIS, and PL results support our hypothesis that the higher photocatalytic activity in the case of ZnO-TiO_2_-CNFs was due to the enhanced conductivity and charge separation.

## 4. Conclusions

We have successfully prepared ZnO-TiO_2_-CNFs composite photocatalyst via electrospinning and hydrothermal methods. The morphological investigation showed that crystalline ZnO particles were well decorated on the TiO_2_-CNFs. The enhanced photocatalytic property in the ZnO-TiO_2_-CNFs composite system was due to the reduced electron-hole recombination rate and adsorption property. From the obtained results, we believe that the as-prepared ZnO-TiO_2_-CNFs composite photocatalyst can be considered as a potential candidate in wastewater treatment and air purification. Furthermore, this work opens a new possibility in the designing of TiO_2_-based composites and promoting their applications in environmental applications.

## Supplementary Materials

The following are available online at https://www.mdpi.com/2079-4991/10/10/1960/s1, Figure S1: TEM-EDX of ZnO TiO_2_ CNFs composite; Figure S2: Cyclic performance of the TiO_2_ NFs and ZnO-TiO_2_-CNFs photocatalysts up to three successive cycles.
