# Supplementary material for: Synthesis and Characterization of ZnO-TiO2/Carbon Fiber Composite with Enhanced Photocatalytic Properties
Source: Nanomaterials (Basel). 2020 Oct 1;10(10):1960. doi: 10.3390/nano10101960 (PMC7600166; doi:10.3390/nano10101960)
Supplement: Supplementary file 1 [file nanomaterials-10-01960-s001.pdf]

nanomaterials

Supporting Information

Synthesis and Characterization of $ZnO-TiO_2$/Carbon Fiber Composite with Enhanced Photocatalytic Properties

Bishweshwar Pant [1], Gunendra Prasad Ojha [1], Yun-Su Kuk [2], Oh Hoon Kwon [3], Yong Wan Park [3] and Mira Park [1,*]

[1] Carbon Composite Energy Nanomaterials Research Center, Woosuk University, Wanju-Gun, Jeollabuk-do 55338, Republic of Korea; bisup@jbnu.ac.kr (B.P.); gpojha10@gmail.com (G.P.O.)

[2] Korea Institute of Carbon Convergence Technology (KCTECH), Jeonju 54853, Republic of Korea; yunsu@kctech.re.kr

[3] Research and Development Division, Korea Institute of Convergence Textile, Iksan 54588, Republic of Korea; simulation@kictex.re.kr (O.H.K.); pywspirit@kictex.re.kr (Y.W.P.)

* Correspondence: wonderfulmira@woosuk.ac.kr

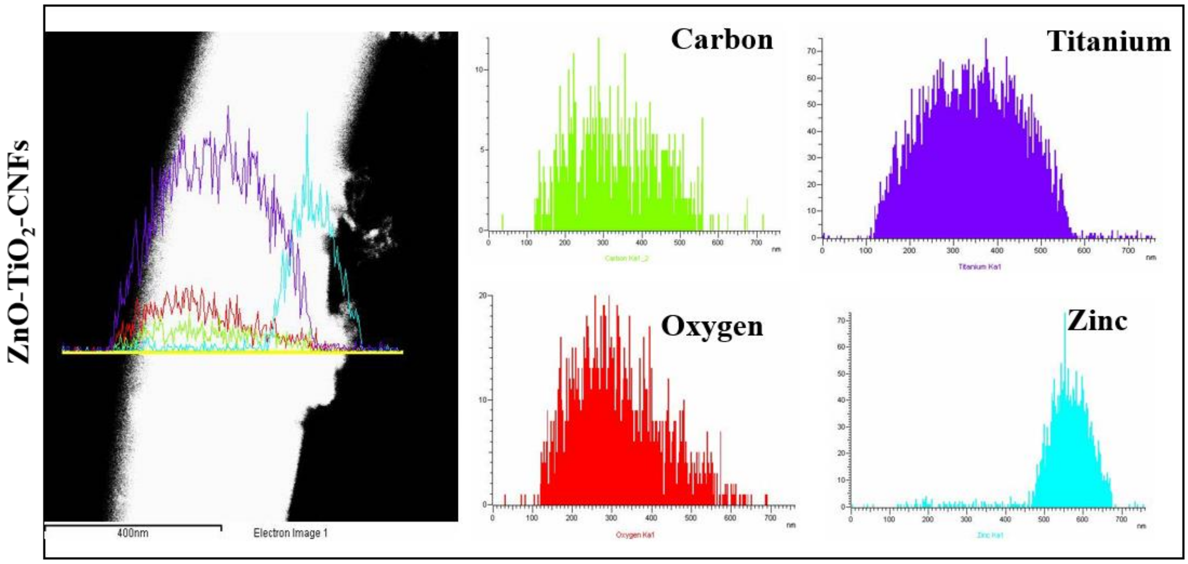

Figure S1. TEM-EDX of ZnO-TiO2-CNFs composite.

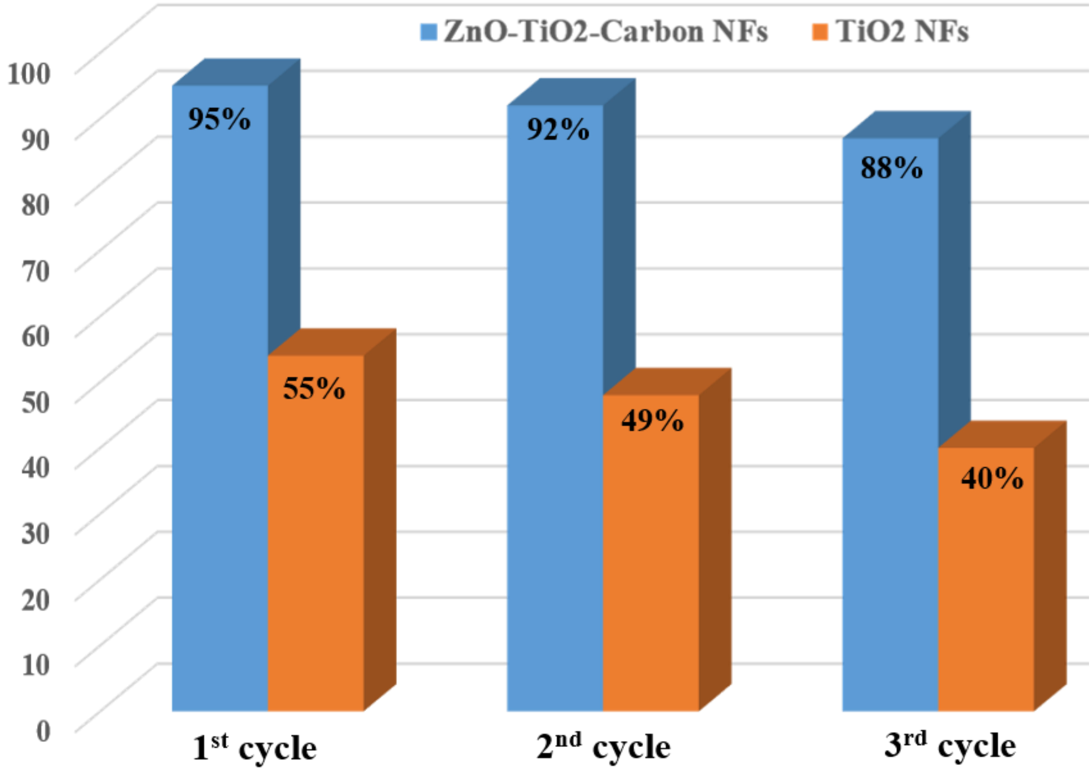

Figure S2. Cyclic performance of the TiO_2 NFs and ZnO-TiO_2-CNFs photocatalysts up to three successive cycles.
